# Adjusting cryodiluent composition for improved post-thaw quality of rabbit spermatozoa

**DOI:** 10.1371/journal.pone.0175965

**Published:** 2017-04-20

**Authors:** Sally E. Hall, Cameron Negus, Danielle Johinke, Roslyn Bathgate

**Affiliations:** Faculty of Veterinary Science, The University of Sydney, Sydney, NSW, Australia; Universidade Federal do Parana, BRAZIL

## Abstract

Improved fertility following artificial insemination with frozen-thawed spermatozoa would offer rabbit producers faster genetic improvement. Previous work investigating cryoprotectants for rabbit spermatozoa have reported inconsistent results. Semen was collected from three rabbit bucks by artificial vagina and frozen using a standard procedure with varied cryodiluent components. Post-thaw analysis encompassed motility, sperm kinematic parameters and acrosome and membrane integrity. Spermatozoa were evaluated at 0, 2 and 4 h after thawing. Experiment 1 compared diluents with 3.5% dimethyl sulfoxide (DMSO), 1.5% acetamide, 1.75% DMSO + 0.75% acetamide or 3.5% DMSO + 1.5% acetamide. The treatment that resulted in the highest post-thaw motility (P<0.001) and acrosome integrity (P<0.001) was DMSO alone. Experiment 2 compared 3.5, 7 and 10% DMSO in the cryodiluent. The best post-thaw sperm motility (P<0.001) and linearity (P = .002) was in 3.5% DMSO, while 10% DMSO afforded higher acrosome/membrane integrity at this last time point (P<0.05). Experiment 3 varied the cryodiluent to contain either 9 or 17% egg yolk or 9 or 17% low density lipoproteins extracted from whole egg yolk. The treatment with the best post-thaw result was 17% egg yolk (motility, P = 0.01; acrosome/membrane integrity, P<0.001). Experiment 4 compared different carbohydrates in the cryodiluent; 50 mM glucose (TCG), 25 mM glucose with 25 mM sucrose (TCGS low), or 50 mM glucose with 50 mM sucrose (TCGS high). When data were pooled across time points, TCG had significantly higher motility than TCGS high (P = 0.021), but was not different from TCGS low. However, TCG had fewer spermatozoa with intact acrosomes and membranes than both TCGS low and TCGS high (P = .002). Put together, these results indicate that the best cryodiluent for rabbit spermatozoa frozen under the conditions used in this paper is with 7% DMSO and 17% egg yolk in a base medium containing 25 mM glucose and 25 mM sucrose.

## Introduction

Due to a high feed conversion rate [1], rabbits remain an important means by which to meet the nutritional demands of the increasing world population. This has led to a demand for a breeding program focused on improving genetic selection for prolificacy, meat quality and continued feed efficiency [2]. To this end, semen from superior males with desired traits must be transported in such a manner that sperm functionality is maintained. While it would be ideal to share genetics internationally, importation of semen between countries is restricted due to quarantine requirements [3]. One possible means of overcoming these quarantine restrictions is development of commercially viable sperm cryopreservation protocols, to enable health testing of males prior to spermatozoa being introduced into a new environment.

In most countries, the rabbit industry currently relies on artificial insemination of fresh or chilled spermatozoa stored for no more than three days [4] but more frequently up to 12 hours [5]. This is because the cryopreservation of rabbit semen has so far shown to result in poor post-thaw sperm quality, leading to low pregnancy rates and litter sizes [6]. There is a paucity of research into defining a cryopreservation protocol for rabbit spermatozoa that achieves commercially acceptable pregnancy rates; to this end, the present study aimed to remedy this by investigating the effect of a variety of cryoprotectants and carbohydrates in the cryodiluent.

It seems clear that, unlike the spermatozoa of other species which are able to survive freeze-thawing well in the presence of glycerol, rabbit spermatozoa survive better when a highly permeable penetrating cryoprotectant is used (reviewed by [6]). Two such compounds are dimethyl sulfoxide (DMSO) and acetamide. It has previously been found that using DMSO as the cryoprotectant results in reasonable post-thaw motility after cryopreservation of rabbit sperm [7]. Interestingly, there appears to be a strong interaction between egg yolk and DMSO. This has been demonstrated by some authors concluding that better post-thaw results are achieved in cryodiluent with low concentrations of DMSO (≤4%) in the presence of egg yolk [8], compared with another group showing DMSO at high concentrations (≥10%) gave better post-thaw sperm quality in the absence of egg yolk [9].

Acetamide has also been used previously for rabbit sperm cryopreservation, reportedly providing better cryoprotection of spermatozoa than the other cryoprotectants with which it was compared, such as glycerol, DMSO and ethylene glycol [10, 11]. However, these comparisons have been rudimentary, with little attempts to investigate a dose response or to consider interactions with other diluent components.

Egg yolk has been routinely used in sperm cryodiluents for many years, due to its role in stabilising sperm membranes and reducing damage to sperm membranes during cooling. The search for a substitute has been based on the desire to find a non-biological hazardous component that will afford the same protection. The use of extracted low-density lipoproteins (LDL) in sperm cryodiluents has been reported as an alternative to whole egg yolk as far back as 1976 [12], based on the proposition that it is the protective component of yolk. More recent studies have shown higher post-thaw motility when spermatozoa are frozen in cryodiluents supplemented with LDL compared with whole egg yolk supplementation (bull; [13], boar; [14]). The post-thaw quality of rabbit spermatozoa frozen in cryodiluent with 16% DMSO containing either whole yolk or LDL have been compared [15]. These authors concluded that freezing in diluent with 10% (final concentration) LDL resulted in higher post-thaw motility and viability than that achieved by freezing in diluent with 15% egg yolk. However, these studies did not consider the previously discussed interaction between high concentrations of DMSO and egg yolk.

Another means of affording cryoprotection to rabbit spermatozoa has been the use of high concentrations of sugar. These compounds are thought to protect cells by modulating dehydration and so preventing the formation of intercellular ice [16]. Additionally, sugar has long been known to interact with the phospholipid membranes to aid with stabilisation by reducing lipid disorder [17]. The difference in the mechanism of action between different types of sugars (such as mono- or di-saccharides) determines the type or localization of the protective impact on spermatozoa. Thus, a comparison of mono- and disaccharides or a combination of both in cryodiluent for rabbit spermatozoa is warranted.

The aim of the present study was to compare the post-thaw quality of rabbit spermatozoa after cryopreservation in cryodiluents containing different cryoprotectants, following modification of protocols that have previously reported high post-thaw motility and viability of rabbit spermatozoa.

## Materials and methods

This study was carried out with the approval of the University of Sydney Animal Ethics Committee (2013/5505). Unless otherwise stated, all chemicals were sourced from Sigma Aldrich (Castle Hill, NSW, Australia).

### Animals

This research was based on multiple donations from three normozoospermic sexually mature rabbits (*Oryctolagus cuniculus*), of mixed breeds of Flemish Giant, Crusader and New Zealand White. This mix of breeds is commonly seen in the Australian rabbit industry. The rabbits were housed in large individual pens, fed a commercial diet and provided water *ad libitum*. The rabbitry was maintained at 25°C and lights were set on an 8 h light:16 h dark cycle.

### Semen collection and evaluation

Ejaculates were collected twice weekly using a teaser doe and artificial vagina (IMV, L’Aigle, France). When present, the gel plug was removed from the ejaculate, and semen colour and volume immediately recorded. Sperm concentration was assessed using a haemocytometer. To be included in the study, ejaculates were required to be at least 400 μL in volume, have a minimum concentration of 300 x 10^6^ spermatozoa, be white in appearance and creamy in consistency. Ejaculates with evidence of urine contamination were excluded from the study.

### Sperm cryopreservation

Ejaculates were diluted with a Tris-citric acid-glucose (TCG) diluent consisting of 313.79 mM Tris, 103.07 mM citric acid, 33.3 mM D-(+)-glucose and 80 mg/L kanamycin; [4], with 17% egg yolk (v/v). The egg yolk in the diluent was clarified by centrifugation at 12,000 × *g* for 30 min at room temperature [18]. After initial assessment, the semen was diluted 1:1 (v:v) with the cryodiluent to be assessed and cooled in a 100 mL water jacket to 15°C over 1 h. The semen was then further cooled to 5°C over 1.5 h after which chilled spermatozoa were packaged into 0.25 mL straws (IMV, L’Aigle, France) sealed with polyvinyl powder (IMV, L’Aigle, France) and frozen by suspension 5 cm above liquid nitrogen for 15 min, followed by plunging into liquid nitrogen for storage. Immediately prior to assessment, straws were thawed by agitation in a 37°C water bath for 40 s. Spermatozoa were released into warmed borosilicate tubes for assessment at 0, 2 and 4 h post-thaw. Samples were maintained at 37°C over the duration of the assessment period.

#### Experiment 1

In the first experiment, four penetrating cryoprotectant concentrations were compared. Each ejaculate was divided into four equal aliquots for dilution in cryodiluent supplemented with either: 3.5% DMSO (DMSO), 1.5% acetamide (ACE), 1.75% DMSO + 0.75% acetamide (DMSO/ACE low) or 3.5% DMSO + 1.5% acetamide (DMSO/ACE high).

#### Experiment 2

In the second experiment, the best cryoprotectant treatment (DMSO alone) from experiment 1 was further investigated to determine an optimal concentration. Ejaculates were divided into three aliquots and frozen in cryodiluent with either 3.5, 7 or 10% DMSO.

#### Experiment 3

The third experiment investigated replacing egg yolk with extracted LDL. The most efficacious diluent from experiment 2 (7% DMSO) was adjusted so the final treatments contained either 9% egg yolk (EY9), 9% LDL (LDL9), 17% egg yolk (EY17) or 17% LDL (LDL17).

Low-density lipoproteins were extracted as described previously [19]. Briefly, equal volumes (1:1) of egg yolk and 0.1 M sodium chloride (NaCl) were combined and the solution mixed for 1 h at 5°C. The solution was then centrifuged at 10,000 × *g* for 45 min at 4°C. The supernatant was decanted and centrifuged for another 45 min at 10,000 × *g* at 4°C.

#### Experiment 4

The fourth experiment investigated the use of different carbohydrates in the diluent. The best cryodiluent from the previous experiment (with 17% EY) was adjusted to contain: 50 mM glucose (control; TCG), a combination of 25 mM glucose and 25 mM sucrose (TCGS low), or a combination of 50 mM glucose and 50 mM sucrose (TCGS high).

### Motility analysis

Motility and sperm kinematics were objectively determined using computer assisted sperm analysis (CASA; IVOS v 12, Hamilton Thorne, Danvers, MA, USA), using the following settings: frame rate– 60 Hz; minimum contrast– 50; low static size gates– 0.35; high static size gates– 3.40; low intensity gates– 0.75; high intensity gates– 1.70; low elongation gates– 0.0; high elongation gates– 70; default cell size– 7 pixels; default cell intensity– 70. Aliquots of 5.5 μL from each sample were placed on pre-warmed slides (Cell Vu, Millennium Sciences Inc., NY, USA; 37°C) and a cover slip was added. Assessment of at least three randomly selected microscopic fields and a minimum of 200 cells per aliquot was conducted to determine motility characteristics. The kinematic parameters evaluated were motility (%), progressive motility (%), average path velocity (VAP, μm/s); straight-line velocity (VSL, μm/s); curvilinear velocity (VCL, μm/s); linearity index (LIN, %) and straightness (STR, %).

### Flow cytometry

Flow cytometry was performed using a FACScan flow cytometer (Becton Dickinson, Franklin Lakes, NJ, USA) with a 488 nm argon ion laser. Emission measurements were made using 530/30 bandpass (green/FL-1), 585/42 bandpass (red/FL-2) and >670 longpass (far red/FL-3) filters. Debris was gated out using a forward scatter/side scatter dot plot, and 10,000 cells were analysed per sample. All data were analysed using CellQuest software (Becton Dickinson). Sperm acrosome integrity and plasma membrane integrity were assessed using dual-staining fluorescein isothiocyanate conjugated peanut agglutinin (FITC-PNA; 4 μg/mL) and propidium iodide (PI; 12 μM), respectively. The sample was incubated for 5 min at 37°C after staining.

### Statistical analyses

Data were analysed using Linear Mixed Model in GenStat (Version 16, VSN International Ltd., Hemel Hempstead, UK) to determine the effects of the treatments, the effect of the time of analysis, and the interaction between treatment and time. The effects of diluent and time were included in the fixed model, with replicate/diluent as the random model. For all analyses, significance was defined as P<0.05.

## Results

### Experiment 1

**Fig 1:**
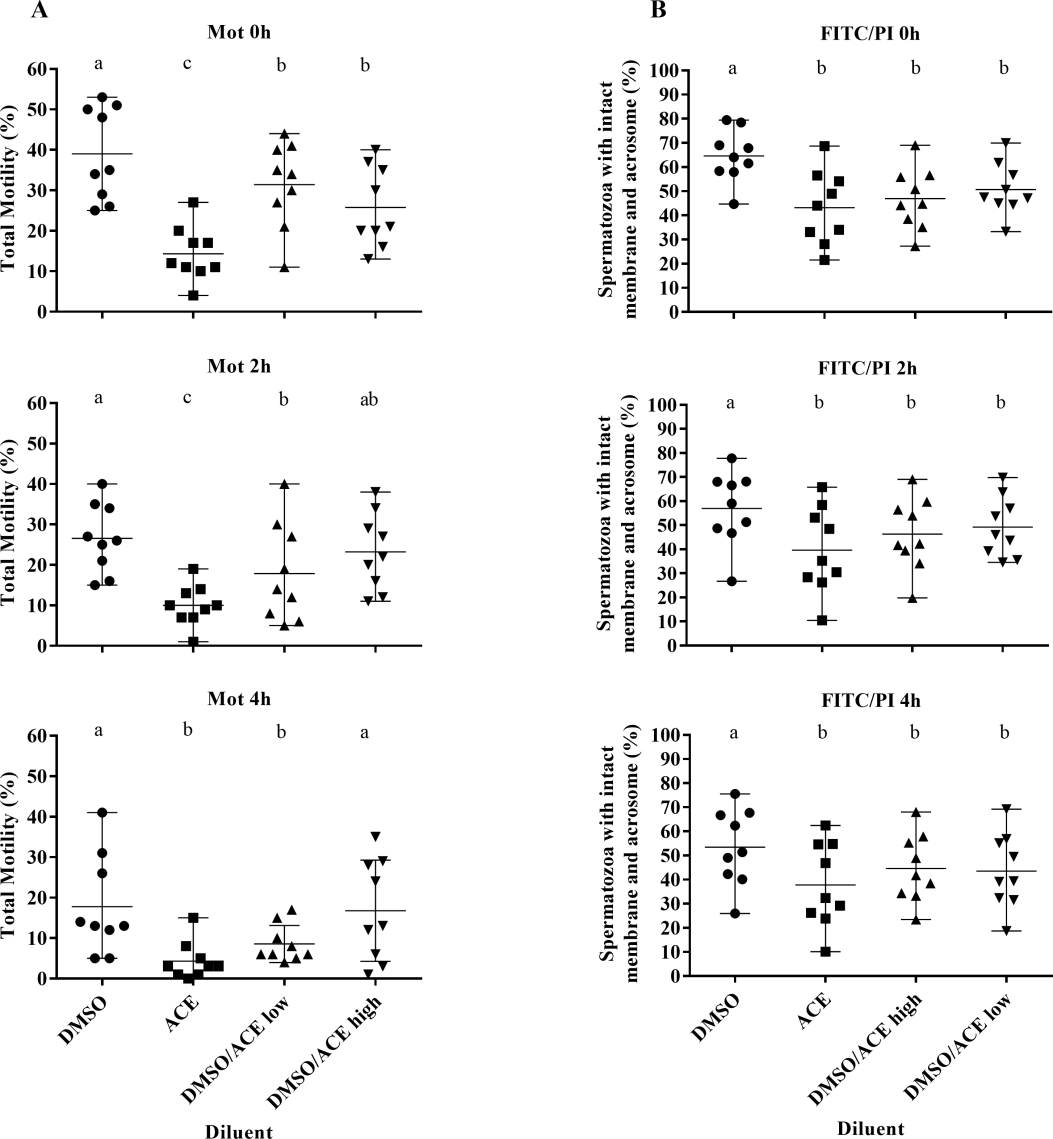
Total motility and flow cytometric results from experiment 1. Post-thaw motility (A) and proportion of spermatozoa with intact membranes and acrosomes (B) at 0, 2 or 4 hours after thawing with incubation at 37°C of rabbit spermatozoa frozen in diluent supplemented with 3.5% DMSO (●), 1.5% acetamide (■), 3.5% DMSO and 1.5% acetamide (▲) or 1.75% DMSO and 0.75% acetamide (▼). Each point represents datum from a replicate and the horizontal lines represent range around the mean. Superscripts represent significant differences within time points between treatment groups.

There were significant differences (P<0.001) in total motility between spermatozoa frozen in different extenders at each time point post-thaw, as shown in Fig 1A. Generally, diluents with some level of DMSO better protected the motility of spermatozoa during cryopreservation. Kinematic parameters were similar between treatments at each time point, with the exception of VAP, where the ACE treatment was significantly poorer than the other treatments at 2h post-thaw and LIN, where the DMSO treatment returned greater linearity than the other treatments at each time point (p = 0.036; Table 1). Supplementation with DMSO alone returned a significantly (P<0.001) higher proportion of spermatozoa with intact membrane/intact acrosomes at each time point post-thaw compared with diluent containing acetamide (Fig 1B).

**Table 1:** Motility kinematics from experiment 1.

**0h**	**DMSO**	**ACE**	**DMSO/ACE low**	**DMSO/ACE high**
**VAP (**μ**m/s)**	53.0±1.9	48.3±1.8	53.6±2.1	53.0±2.2
**VSL (**μ**m/s)**	42.7±1.5	38.4±1.5	39.9±1.1	39.6±1.2
**VCL (**μ**m/s)**	96.0±3.2	97.9±3.0	104.3±3.7	102.6±3.
**LIN (%)**	47.0±2.6^a^	40.6±1.1^b^	39.7±0.8^b^	39.9±1.2^b^
**STR (%)**	81.4±1.2	79.8±1.2	76.3±1.2	76.1±1.5
**2h**	**DMSO**	**ACE**	**DMSO/ACE low**	**DMSO/ACE high**
**VAP (**μ**m/s)**	52.9±1.4^a^	44.1±5.8^b^	51.1±1.6^a^	51.0±1.3^a^
**VSL (**μ**m/s)**	45.0±1.3	34.8±4.5	40.5±1.4	40.1±1.2
**VCL (**μ**m/s)**	97.8±4.9	88.5±11.6	103.0±3.7	102.6±3.5
**LIN (%)**	47.7±1.8^a^	36.0±4.7^c^	41.2±1.5^b^	40.1±1.2^b^
**STR (%)**	85.0±0.7	70.4±8.9	79.2±1.0	78.8±0.8
**4h**	**DMSO**	**ACE**	**DMSO/ACE low**	**DMSO/ACE high**
**VAP (**μ**m/s)**	47.9±1.3	47.1±1.6	44.4±1.4	47.0±1.4
**VSL (**μ**m/s)**	40.6±1.5	38.2±1.8	34.6±1.1	36.0±1.1
**VCL (**μ**m/s)**	90.0±3.0	95.8±4.9	96.3±4.9	98.2±4.9
**LIN (%)**	46.4±1.9^a^	44.9±2.3^a^	37.6±1.6^b^	38.2±2.1^b^
**STR (%)**	84.7±0.8	76.7±5.5	77.8±1.1	75.9±1.7

Mean ± SEM kinematic scores at 0, 2 or 4 hours after thawing with incubation at 37°C of rabbit spermatozoa frozen in diluent supplemented with 3.5% DMSO (DMSO), 1.5% acetamide (ACE), 3.5% DMSO and 1.5% acetamide (DMSO/ACE high) or 1.75% DMSO and 0.75% acetamide (DMSO/ACE low).

^a,b,c^. Superscripts represent significant differences within time points of each parameter. VAP–average path velocity; VSL–straight line velocity; VCL–curvilinear velocity; LIN–linearity (VSL/VCL); STR–straightness (VSL/VAP).

### Experiment 2

**Fig 2:**
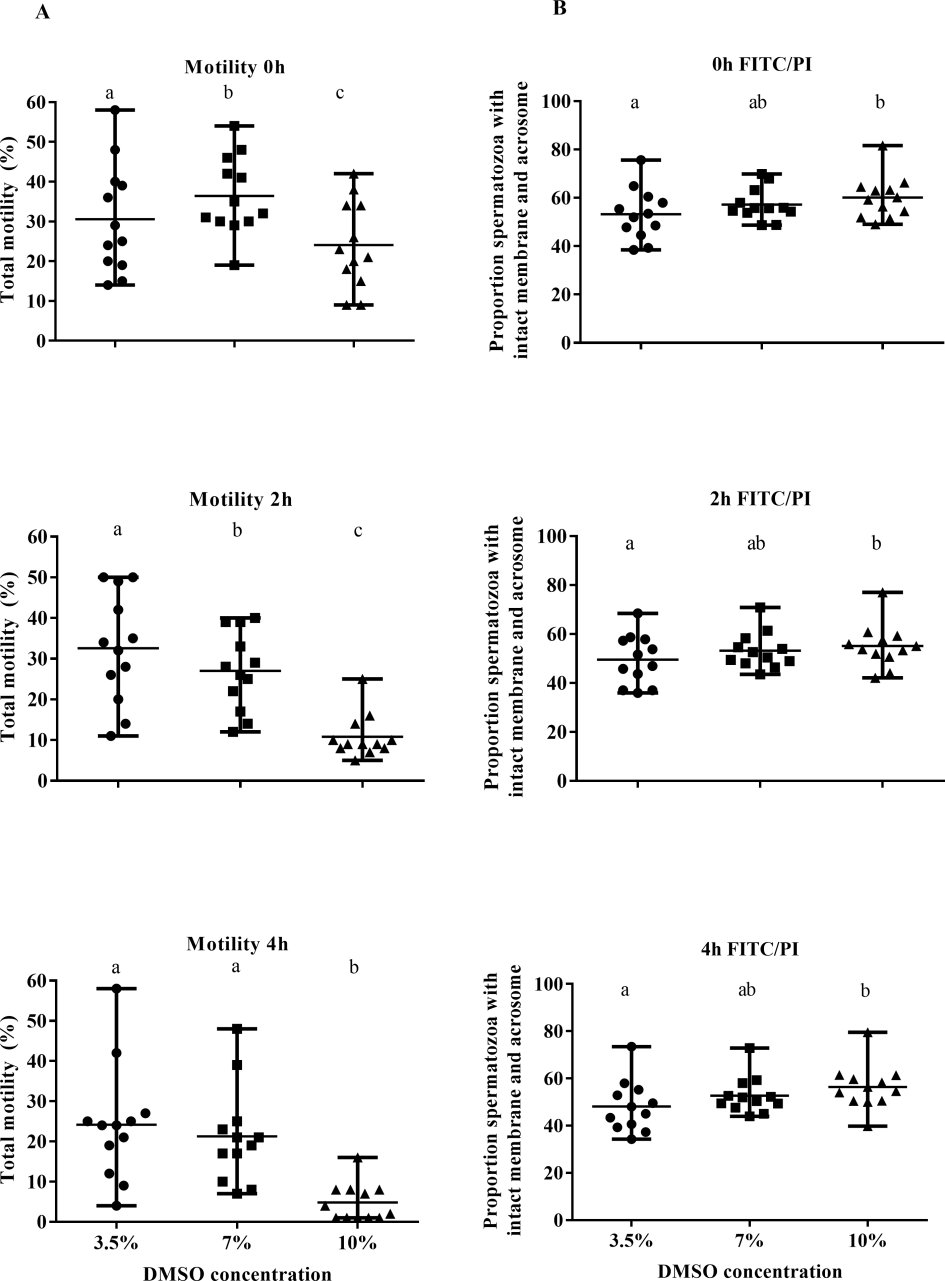
Total motility and flow cytometric results from experiment 2. Post-thaw motility (A) and proportion of spermatozoa with intact membranes and acrosomes (B) at 0, 2 or 4 hours after thawing with incubation at 37°C of rabbit spermatozoa frozen in diluent supplemented with 3.5% (●), 7% (■) or 10% (▲) DMSO. Each point represents datum from a replicate and the horizontal lines represent range around the mean. Superscripts represent significant differences within time points between treatment groups.

The cryoprotective effects of varying concentrations of DMSO in a Tris-based diluent on the motility of frozen-thawed rabbit spermatozoa are illustrated in Fig 2A. Immediately after thawing, spermatozoa frozen in cryodiluent with 7% DMSO had a significantly higher motility than that frozen in either 3.5 or 10% DMSO (p<0.001). However, at two hours post-thaw, spermatozoa frozen with 3.5% DMSO recorded significantly higher motility than those frozen in 7% or 10% DMSO (p<0.001); while at four hours post-thaw, both 3.5% and 7% DMSO produced a significantly higher progressive motility than 10% DMSO (p<0.001).

With respect to the kinetic parameters VAP, VSL and VCL, patterns of results similar to overall motility were observed (Table 2). Samples diluted with 3.5% DMSO had significantly higher post-thaw mean values of VAP, VSL, and VCL than those diluted with 10% DMSO at each time point post-thaw (P<0.001). There were no significant differences on LIN and STR found between varied DMSO concentrations.

**Table 2:** Motility kinematics from experiment 2.

**0h**	**3.5% DMSO**	**7% DMSO**	**10% DMSO**
**VAP (**μ**m/s)**	45.1±0.9^a^	42.7±0.6^a^^b^	40.1±0.7^b^
**VSL (**μ**m/s)**	34.5±0.8^a^	33.1±0.8^a^^b^	31.4±0.6^b^
**VCL (**μ**m/s)**	99.5±1.3^a^	94.1±1.3^a^^b^	89.5±1.4^b^
**2h**	**3.5% DMSO**	**7% DMSO**	**10% DMSO**
**VAP (**μ**m/s)**	51.1±1.0^a^	48.0±1.5^b^	39.1±1.0^c^
**VSL (**μ**m/s)**	41.6±0.7^a^	39.7±1.3^a^	40.5±1.4^b^
**VCL (**μ**m/s)**	110.6±2.0^a^	107.2±2.1^a^	92.8±2.0^b^
**4h**	**3.5% DMSO**	**7% DMSO**	**10% DMSO**
**VAP (**μ**m/s)**	48.7±1.6^a^	42.5±1.4^b^	36.6±1.0^c^
**VSL (**μ**m/s)**	39.7±1.3^a^	34.6±1.3^b^	28.6±0.8^c^
**VCL (**μ**m/s)**	107.2±3.6^a^	96.4±2.5^b^	83.8±3.2^c^

Mean ± SEM kinematic scores at 0, 2 or 4 hours after thawing with incubation at 37°C of rabbit spermatozoa frozen in diluent supplemented with 3.5, 7 or 10% DMSO.

^a, b, c.^ Superscripts represent significant differences within time points of each parameter. VAP–average path velocity; VSL–straight line velocity; VCL–curvilinear velocity.

In contrast to the motility parameters, higher concentrations of DMSO proved to be more beneficial at preserving sperm membranes and acrosomes (Fig 2B). Diluents containing 10% DMSO produced significantly higher proportions of intact membranes after thawing compared with 3.5% DMSO at each measured time point (P<0.05). Diluents with 7% did not significantly differ in percentage of intact membranes and acrosomes from either of the other two DMSO concentrations at each time point.

#### Experiment 3

**Fig 3:**
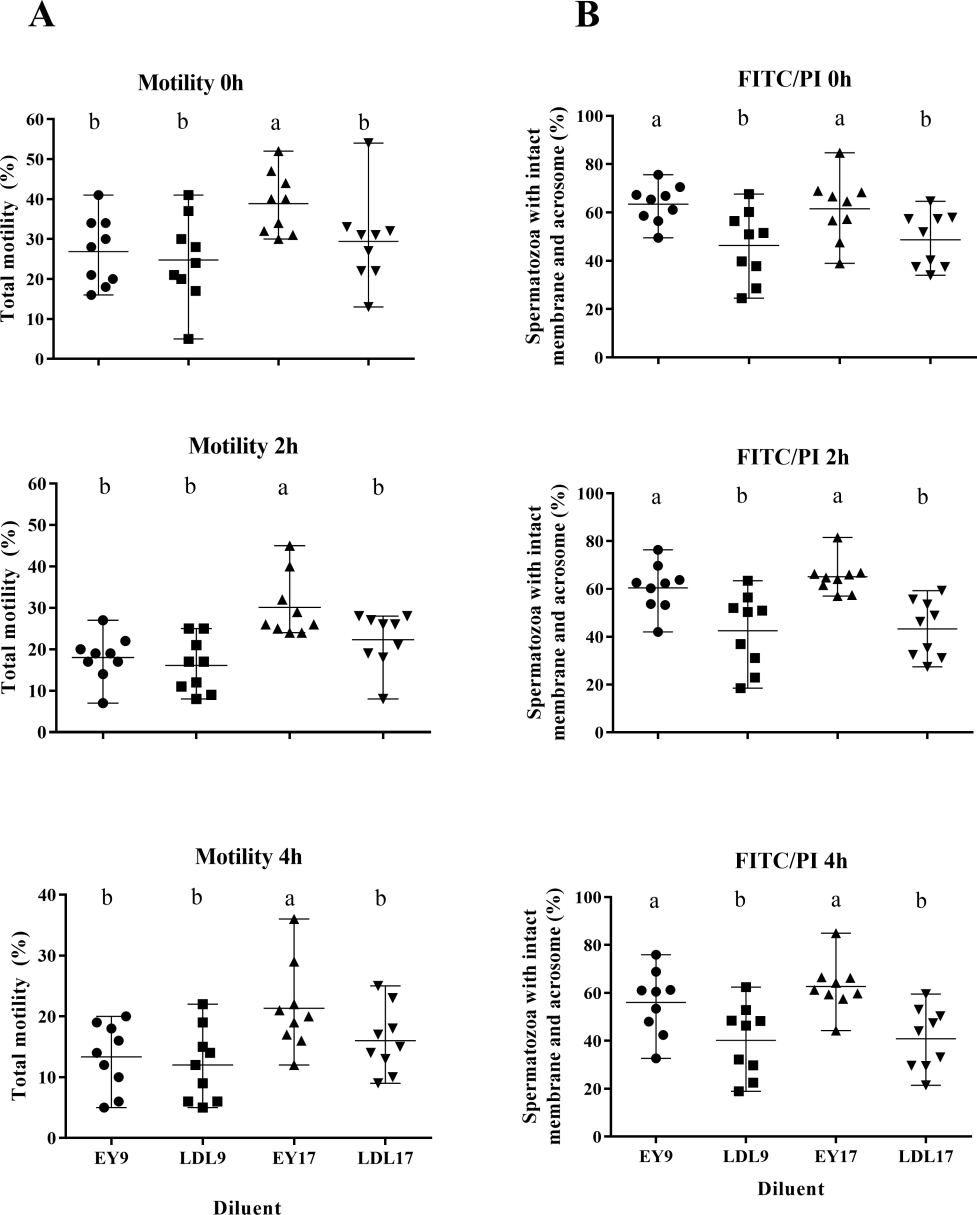
Total motility and flow cytometric results from experiment 3. Post-thaw motility (A) and proportion of spermatozoa with intact membranes and acrosomes (B) at 0, 2 or 4 hours after thawing with incubation at 37°C of rabbit spermatozoa frozen in diluent supplemented with 9% egg yolk (EY9; ●), 9% LDL (LDL9; ■), 17% egg yolk (EY17;▲) or 17% LDL (LDL17; ▼). Each point represents datum from a replicate and the horizontal lines represent range around the mean. Superscripts represent significant differences within time points between treatment groups.

Sperm frozen in EY17 had a significantly higher (P = 0.01) total motility at all time points post-thaw than spermatozoa frozen in other diluents (Fig 3A). All other motility parameters assessed were similar between treatments, with the exception of STR, with spermatozoa frozen in either EY17 or LDL17 exhibiting a higher degree of STR (76.8 ± 1.04% and 75.9 ± 0.98%, respectively) than that frozen in EY9 or LDL9 (70.1 ± 1.41% and 71.7 ± 1.33%, respectively) at 0 h post-thaw (p = 0.0004; Table 3).

**Table 3:** Motility kinematics from experiment 3.

**0h**	**EY9**	**LDL9**	**EY17**	**LDL17**
**VSL (**μ**m/s)**	39.1±1.4	40.2±1.3	38.3±0.8	40.1±1.5
**VAP (**μ**m/s)**	57.5±1.8	57.1±2.2	50.7±1.3	53.6±2.0
**STR (%)**	70.1±1.4^a^	71.7±1.3^a^	76.8±1.0^b^	75.9±1.0^b^
**2h**	**EY9**	**LDL9**	**EY17**	**LDL17**
**VSL (**μ**m/s)**	41.8±0.5	40.3±1.6	39.7±1.5	42.4±0.8
**VAP (**μ**m/s)**	53.4±0.9	51.4±1.6	49.8±0.8	52.8±1.0
**STR (%)**	78.7±0.8	78.3±0.7	80.0±1.7	80.2±0.9
**4h**	**EY9**	**LDL9**	**EY17**	**LDL17**
**VSL (**μ**m/s)**	37.2±1.5	37.4±1.2	38.4±0.6	39.8±1.2
**VAP (**μ**m/s)**	46.9±1.7	47.2±1.4	48.3±0.6	49.3±1.1
**STR (%)**	79.6±0.9	79.3±0.9	79.7±1.2	80.3±0.9

Mean ± SEM kinematic scores at 0, 2 or 4 hours after thawing with incubation at 37°C of rabbit spermatozoa frozen in diluent supplemented with 9% egg yolk (EY9), 9% LDL (LDL9), 17% egg yolk (EY17) or 17% LDL (LDL17).

^a, b.^ Superscripts represent significant differences within time point of each parameter. VSL–straight line velocity; VAP–average path velocity; STR–straightness (VSL/VAP).

The flow cytometric assessment indicated that samples frozen in diluent containing egg yolk maintained a significantly higher (P<0.001) proportion of spermatozoa with intact membranes and acrosomes than those frozen in diluent containing LDL, at all time points post-thaw (Fig 3B).

### Experiment 4

**Fig 4:**
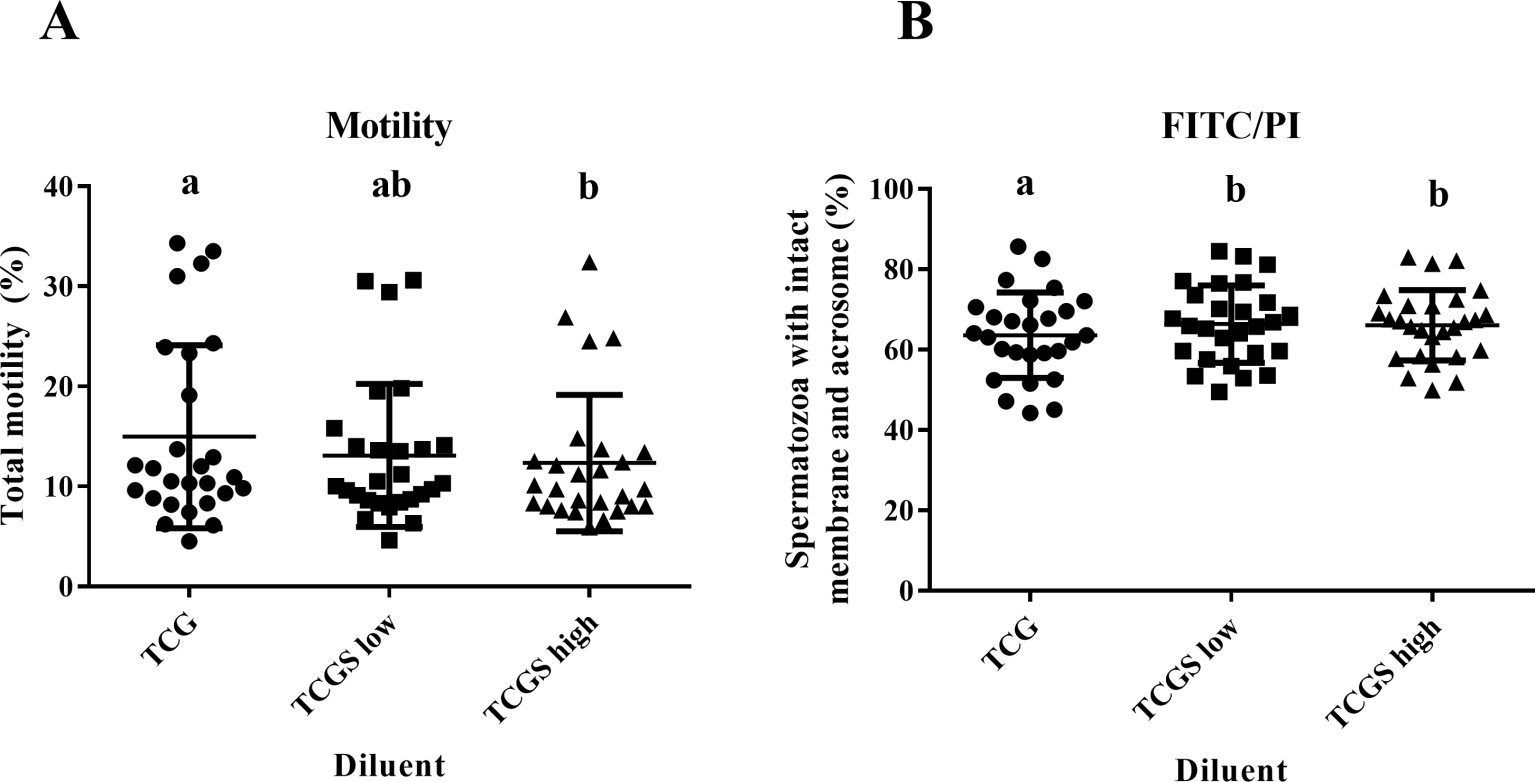
Total motility and flow cytometric results from experiment 4. Post-thaw motility (A) and proportion of spermatozoa with intact membranes and acrosomes (B) from data pooled across 0, 2 or 4 hours post-thaw with incubation at 37°C of rabbit spermatozoa frozen in diluent containing 50 mM glucose as the sole carbohydrate source (Control; TCG; ●), a combination of 25 mM glucose and 25 mM sucrose (TCGS low; ■), or a combination of 50 mM glucose and 50 mM sucrose (TCGS high;▲). Each point represents datum from a replicate and the horizontal lines represent range around the mean. Superscripts represent significant differences within time points between treatment groups.

The quality of sperm after cryopreservation and incubation with various sugar components in the diluents is detailed in Fig 4A and 4B. There was no interaction between time and experimental group, but when considered across all time points the spermatozoa frozen in TCG exhibited higher total motility than that frozen in TCGS high (P = 0.021), but neither of these differed from the motility scores of spermatozoa frozen in TCGS low. There were no significant differences seen other than CASA-assessed parameters of post-thaw sperm quality between groups. However, when considering results across time points, freezing in TCG resulted in fewer live spermatozoa with intact acrosome than when freezing in both TCGS low and TCGS high (P = 0.002).

## Discussion

This study demonstrated the potential for improved quality of post-thaw rabbit sperm quality to be achieved with minor adjustments of cryoprotectant type and concentration. The improvements in sperm parameters and viability observed in this study suggest that, with further study investigation, commercially viable protocols for cryopreservation of rabbit semen may be developed.

Sperm freezing protocols in many species typically use a fast freezing rate (approximately -10°C/min) at temperatures below 5°C with glycerol as the main cryoprotectant. However, previous research has demonstrated that rabbit spermatozoa have a higher survival rate with a relatively slow cooling rate and a cryoprotectant that permeates rapidly, likely due to a lower water permeability relative to other domestic species [6, 11, 20, 21]. DMSO and acetamide are candidates for this role, as they have low molecular weights and so can permeate the cell rapidly [6, 22].

The post-thaw parameters observed in this study suggest that use of DMSO as a cryoprotectant for rabbit spermatozoa is preferable to acetamide. This is despite previous studies finding higher post-thaw motility of rabbit spermatozoa frozen in diluent containing acetamide [10], or no difference between DMSO and acetamide when considering total motility and membrane integrity only [11]. Perhaps the conflicting results may have arisen from differences in the freezing rates used as the straws in this study were frozen 6 cm above liquid nitrogen, whereas previous studies have implemented a distance of 4 cm [11] or 10 cm above liquid nitrogen [10]. Due to the differences in penetration rates between DMSO and acetamide, it would be expected that their protective capacity is more suited to dissimilar freezing rates. This could be confirmed by altering freezing rates when cryopreserving rabbit spermatozoa. Future work in this area may provide insight into optimal freezing rates, where a two-step freezing protocol may be investigated.

Consideration of the kinematic measurements in this experiment showed that spermatozoa frozen in the 3.5% DMSO displayed a significantly higher proportion of spermatozoa with linear motility (LIN) than all other groups tested at all time points. This parameter that has previously been shown to be correlated with high fertility in bulls [23] and rabbits [24]. Thus, the decision was made to use the diluent containing DMSO only for further investigation in Experiment 2.

Previous studies have also indicated a relationship between the concentration of egg yolk and other cryoprotectants, including DMSO in cryodiluents. DMSO has been shown to be most effective at preserving rabbit sperm quality post-thaw at high concentrations in the absence of egg yolk [6, 20]. With lower concentrations of DMSO, the beneficial effects of higher egg yolk concentration become detectable [8, 25]. However, these results must be interpreted with caution, as there is some evidence that egg yolk has a stimulatory effect on sperm motility [26].

Interestingly, the CASA parameters VAP, VSL and VCL showed a clear and consistent concentration effect of DMSO across all time points, with the lower DMSO concentration scoring higher values. While these have not demonstrated a correlation with fertility in rabbits [24], faster VSL values correlated to higher fertility in bulls [27] and more rapid VAP and VSL scores have been related to higher total number of offspring born in pigs [28]. In an effort to balance the motility results (where 3.5% was best) and the acrosome and membrane integrity results, the decision to continue with 7% DMSO for experiment 3 was made.

The results from Experiment 3 demonstrated a clear benefit to freezing in diluents supplemented with egg yolk compared with LDL supplementation, in terms of the proportion of spermatozoa with both intact acrosomes and membranes. This infers there are components in egg yolk other than LDL that aid in the protection of spermatozoa against cold shock. Alternatively, as LDL make up only 2/3 of whole yolk, perhaps a lower concentration of LDL in the diluent would have provided better post-thaw results as found by researchers in dog semen [29].

The final aspect of cryodiluents that was investigated in this study was the sugar type. Carbohydrates act not only as an energy source in cryodiluents but also play a role in maintaining osmolarity and provide cryoprotection by obstructing intracellular ice formation through cellular dehydration [30]. It has been demonstrated in other species that disaccharides provide better cryoprotection to spermatozoa than monosaccharides (boar, [31]; mouse, [32]). While the reason behind this has not been elucidated, one proposed mechanism is the higher osmotic pressure exerted by the disaccharides, more effectively reducing the formation of intracellular ice crystals [33]. Alternatively, it has been suggested that spermatozoa from each species show varying levels of cryosurvival with different saccharides in the diluent [31] and perhaps the combination of monosaccharides that comprise the disaccharide may influence the outcome. The results of this study better support the former hypothesis, as the addition of a disaccharide was beneficial to the cryosurvival of rabbit spermatozoa, but the higher osmotic pressure from doubling the concentration of carbohydrates in the diluent did not improve the post-thaw quality of the spermatozoa. Sucrose is a bonding of glucose and fructose, so it would be of interest to investigate the effects of other carbohydrate combinations in the cryopreservation of rabbit spermatozoa to determine which provides better cryoprotection. Authors that have investigated this previously have suggested that freezing in a HEPES-based diluent containing acetamide and trehalose (composed of two glucose units) was preferable to that containing raffinose (a trisaccharide of glucose, galactose and fructose; [10]). Other authors compared glucose, lactose and sucrose-supplemented Tris-based diluents (with DMSO as the cryoprotectant and without egg yolk) and concluded that sucrose was the carbohydrate of choice under these conditions [34]. While in a different study, the same authors concluded that in a Tris-based diluent with or without egg yolk (and with varying concentrations of DMSO as the cryoprotectants), sucrose was preferable over glucose, lactose (a galactose-glucose disaccharide) and maltose (a glucose-glucose disaccharide, bonded differently to the trehalose pair). Put together, these data suggest that a combination of carbohydrates is preferred for rabbit spermatozoa cryopreservation–perhaps what remains to be clarified is whether they are best made available as a monosaccharide, a disaccharide or even a trisaccharide and with what bond unit within the polysaccharide.

In conclusion, this study demonstrated that a diluent for the cryopreservation of rabbit spermatozoa needs to be optimised according to the concentrations of penetrating and non-penetrating cryoprotectants included in the diluent. While further investigation of breed differences is warranted, the results from this study indicate t that DMSO at 7% concentration in a TCGS low diluent containing 17% egg yolk was found to be the most efficacious at cryopreserving rabbit sperm, with respect to post-thaw motility and viability.
